# Incidence and risk factors for sepsis in surgical patients: a cohort study

**DOI:** 10.1186/cc10165

**Published:** 2011-06-22

**Authors:** AAFS Georgeto, ACGP Elias, MT Tanita, CMC Grion, LTQ Cardoso, P Verri, CFF Veiga, ÁRG Barbosa, AZ Dotti, T Matsuo

**Affiliations:** 1 Hospital Universitário de Londrina, Universidade Estadual de Londrina, Londrina - PR, Brazil

## Introduction

Surgical patients are vulnerable to infectious complications during hospitalization due to several factors. Sepsis seems to be a common complication in the postoperative period, and prompt recognition combined with early interventions is an effective way of reducing mortality in this condition.

## Objective

To evaluate risk factors for sepsis in surgical patients admitted to the intensive care unit (ICU).

## Methods

Prospective data collected from a cohort of surgical patients from January 2005 to December 2007. We analyzed the incidence of sepsis and certain variables from the preoperative, intraoperative and postoperative period as risk factors for sepsis.

## Results

We studied 648 surgical patients. The mortality rate was 19.3% and mean age was 53.2 ± 18.8 years. The incidences of severe sepsis and septic shock were 6.6% and 12.7%, respectively. Multivariate analysis showed that the following variables were associated with sepsis: urgent surgery (OR = 6.92, 95% CI = 4.34 to 11.03), emergency surgery (OR = 5.36, 95% CI = 2.86 to 10.05), POSSUM physiologic variables (OR = 1.03, 95% CI = 1.01 to 1.06), POSSUM surgical variables (OR = 1.09, 95% CI = 1.05 to 1.13), mechanical ventilation (OR = 7.20, 95% CI = 3.78 to 13.71) and Sequential Organ Failure Assessment at ICU admission (OR = 1.13, 95% CI = 1.05 to 1.22).

## Conclusion

The present study detected a high incidence of infectious complications in surgical patients that resulted in high mortality rates. Risk factors associated with sepsis during the perioperative period were easily detectable and knowledge of these can be useful for prevention strategies and early identification of complications.

